# T-DNA insertion mutagenesis in *Penicillium brocae* results in identification of an enolase gene mutant impaired in secretion of organic acids and phosphate solubilization

**DOI:** 10.1099/mic.0.001325

**Published:** 2023-04-17

**Authors:** Juntao Zhang, Xiaoge Han, Yang Su, Christian Staehelin, Changchao Xu

**Affiliations:** ^1^​ Guangzhou Institute of Forestry and Landscape Architecture, Guangzhou 510405, PR China; ^2^​ School of Ecological Environment Technology, Guangdong Industry Polytechnic, Nanhai Campus, Foshan 528225, PR China; ^3^​ State Key Laboratory of Biocontrol and Guangdong Key Laboratory of Plant Resources, School of Life Sciences, Sun Yat-sen University, Guangzhou 510275, PR China

**Keywords:** phosphate solubilization, T-DNA insertion, enolase

## Abstract

*Penicillium brocae* strain P6 is a phosphate-solubilizing fungus isolated from farmland in Guangdong Province, China. To gain better insights into the phosphate solubilization mechanisms of strain P6, a T-DNA insertion population containing approximately 4500 transformants was generated by *Agrobacterium tumefaciens *-mediated transformation. The transformation procedure was optimized by using a Hybond N membrane for co-cultivation of *A. tumefaciens* and *P. brocae*. A mutant impaired in phosphate solubilization (named MT27) was obtained from the T-DNA insertion population. Thermal asymmetric interlaced PCR was then used to identify the nucleotide sequences flanking the T-DNA insertion site. The T-DNA in MT27 was inserted into the fourth exon of an enolase gene, which shows 90.8 % nucleotide identity with enolase mRNA from *Aspergillus neoniger*. Amino acid sequence homology analysis indicated that the enolase is well conserved among filamentous fungi and *Saccharomyces cerevisiae*. Complementation tests with the MT27 mutant confirmed that the enolase gene is involved in phosphate solubilization. Analysis of organic acids in culture supernatants indicated reduced levels of oxalic acid and lactic acid for the MT27 mutant compared to the parent strain P6 or the complementation strain. In conclusion, we suggest that the identified enolase gene of *P. brocae* is involved in production of specific organic acids, which, when secreted, act as phosphate solubilizing agents.

## Introduction

Phosphatic fertilizers are widely used in agriculture and forestry, as phosphorus is an essential macronutrient for plant growth and development. While phosphorus can only be absorbed in a soluble form by plant roots, the soil phosphorus utilization efficiency of plants is usually low due to immobilization of phosphate by metal ions, such as calcium, aluminium and iron. Approximately 70–90 % of phosphorus applied to soil is transformed to an insoluble form, which leads to a large use of phosphatic fertilizers in agriculture [1, 2]. However, the excessive use of chemical fertilizers can have negative consequences for soil fertility and the environment [3–5].

Microbes play an important role in soil phosphorus cycling [6]. Studies have found that various bacteria (e.g. *Pseudomonas *, *Enterobacter* and *Bacillus* strains) and fungi (e.g. *Aspergillus*, *Penicillium* and *Trichoderma* strains) can efficiently transform insoluble phosphates to soluble forms [7–9]. Soil inoculation with these phosphate-solubilizing microbes (PSMs) can promote the plant’s biological availability of soil phosphorus. It has been reported that inoculation of soils with PSMs can considerably elevate the level of soluble phosphates and thus improve plant growth in agriculture and forestry. Various well-performing strains have been developed as environmentally friendly biofertilizers [9–11].

Phosphate solubilization mechanism of PSMs is complex. PSMs often secrete different kinds of low-molecular weight organic acids that are known to be involved in the phosphate solubilization process. Furthermore, organic acid secretion-independent mechanisms have been reported, e.g. secretion of phosphatases and phytase [12–15]. Phosphatases and phytases are often involved in solubilization of phosphorus from organic compounds while secreted organic acids mainly play a key role in solubilization of inorganic phosphate [16]. Phosphatases can catalyse the hydrolysis of phosphor-ester or phosphoanhydride bonds and therefore efficiently release phosphate from organic compounds. Environmental inorganic phosphate levels often influence the microbial production of phosphatases. This mechanism is well understood for alkaline phosphatase of *Escherichia coli* [17]. Phytase activity in soils is responsible for releasing phosphorus from phytate, which is present in organic matter. Phosphate solubilization by phosphatases and phytase has been reported for various bacterial and fungal PSMs from different genera [16]. Organic acids, such as gluconic acid, citric acid, oxalic acid and lactic acid, are also often secreted by PSMs. In general, organic acids promote phosphate solubilization by reducing the extracellular pH and by chelating metal ions to release phosphate [6]. For example, increased phosphate solubilization capacity of two *Penicillium* species (*P. bilaji* and *P. fuscum*) was related to decreased extracellular pH [15]. Likewise, the pH of a culture medium from a *Burkholderia cenocepacia* mutant deficient in phosphate solubilization was higher than that from the wild-type bacterium [2].

In bacteria, various genes involved in phosphate solubilization have been identified. For example, Goldstein and Liu [18] found that a cluster of *pqq* genes plays an important role in phosphate solubilization in *Erwinia herbicola *. These genes are involved in synthesis of pyrroloquinoline quinone, a coenzyme of a glucose dehydrogenase required for synthesis of gluconic acid [18, 19]. Sequence analysis of phosphate solubilization-deficient mutants of *Pseudomonas fluorescens* F113 obtained by transposon insertion showed that phosphate solubilization was mainly dependent on *gcd* (glucose dehydrogenase) and *pqq* genes [20]. The ability of fungi to solubilize phosphate is often stronger than that of bacteria, however, the genetic mechanisms underlying phosphate solubilization have hardly been studied in fungi [21].

*Agrobacterium tumefaciens *-mediated transformation (AtMT) has been found to be an important tool for insertional mutagenesis and forward genetics in the model fungus *Saccharomyces cerevisiae* and several filamentous fungi [22–25]. During the AtMT process, transfer DNA (T-DNA) fragments are introduced into eukaryotic host cells and inserted into the nuclear DNA. The T-DNA insertion appears to occur at random and thus allows creation of T-DNA insertion populations [24, 26–30]. Phenotype screening of the transformed populations for a given trait is usually followed by thermal asymmetric interlaced PCR (TAIL-PCR), which allows identification of flanking sequences at T-DNA insertion sites [31, 32]. For example, Huang *et al*. performed an insertional mutagenesis screen for *Aspergillus fumigatus* and identified a gene (*tptA*) that is involved in adaptation to low iron conditions [33]. Cai *et al*. obtained 32 *Colletotrichum gloeosporioides* mutants with pathogenicity defects from an AtMT library and 16 genomic sequences (flanking T-DNA) were identified with the help of TAIL-PCR [26]. Based on such studies, we assumed that AtMT of filamentous fungi would become an efficient tool for identifying genes involved in phosphate solubilization.

Recently, we have isolated *Penicillium brocae* strain P6, a phosphate solubilization fungus from red soil farmland in Guangdong Province, China. *P. brocae* is a new monoverticillate species, which was firstly isolated from coffee berry borers and later observed on the surface of Thai rice grains [34, 35]. Pradeep *et al*. have reported that *P. brocae* can colonize soils well and degrade plasticizers [36]. In our previous study, we found the P6 strain can effectively increase plant available phosphate levels in the soil and promote the growth of *Brassica chinensis* [37]. AtMT of this strain was optimized with respect to spore concentration, co-cultivation time and concentration of acetosyringone while this method was not used to generate a T-DNA insertion population [38]. For construction of a T-DNA insertion population, we aimed to optimize our AtMT protocol for *P. brocae* in other ways. During the AtMT process, *A. tumefaciens* bacteria and fungal spores (conidia) are mixed and co-cultured on membranes that have been placed on the surface of solid media. The type of such co-cultivation membrane can affect the AtMT efficiency of a given fungus. For example, it was reported that Hybond N, Hybond N^+^ or filter paper led to similar AtMT efficiency in *Aspergillus awamori* whereas the use of nitrocellulose and Hybond C membranes caused lower transformation efficiencies [24]. Vijn and Govers found that the use of Hybond N^+^ for AtMT of *Phytophthora infestans* results in two–threefold more transformants in comparison to nylon membranes while this fungus could not grow on nitrocellulose membranes [39]. Filter membranes appear to serve as scaffold material that facilitates the contact between agrobacterial and fungal cells [40]. These reports inspired us to further optimize the AtMT efficiency of the P6 strain by testing different kinds of co-cultivation membranes.

In this study, we found that the use of a Hybond N membrane can increase the AtMT efficiency of *P. brocae* strain P6. A T-DNA insertion population was then generated by the optimized AtMT system, and a subsequent mutant screen resulted in identification of a mutant impaired in phosphate solubilization. Using TAIL-PCR, we found that the T-DNA in this mutant was inserted into an enolase gene. Complementation tests confirmed that the enolase gene is involved in phosphate solubilization. Finally, we report that culture supernatants of the mutant show considerably reduced levels of phosphate, oxalic acid and lactic acid. To our knowledge, this study was the first report of studying genes involved in phosphate solubilization of *Penicillium* sp.

## Methods

### Strains and plasmids

*P. brocae* strain P6 (wild-type, WT) was isolated previously [37] and stored at −80 °C. To obtain T-DNA insertion transformants of P6, *A. tumefaciens* strain AGL-1 harbouring the binary vector pCAMBIA1303-TrpC-Hygro-gpdA-GUS-GFP was used [pCAMBIA1303-TrpC-Hygro-gpdA-GUS-GFP containing a hygromycin resistance gene with a fungal *TrpC* promoter and a GUS (β-glucuronidase)-GFP (green fluorescent protein) fusion gene with a fungal *gpdA* promoter; Fig. S1A, available in the online version of this article]. Strain AGL-1 [41] was kindly provided by Professor Jianfeng Li (Sun Yat-sen University, Guangzhou, China), and the plasmid pCAMBIA1303-TrpC-Hygro-gpdA-GUS-GFP was purchased from Miaolingbio Company (Wuhan, China). The enolase gene mutant of P6 identified in this study was named MT27.

For complementation tests, the enolase gene of *P. brocae* P6 (GenBank accession number ON455119) driven by the *gpdA* promoter was expressed in the MT27 mutant. The plasmids pBARGPE1-EGFP [containing an enhanced GFP sequence with a *gpdA* promoter and a bialaphos resistance gene (*bar*) with a *TrpC* promoter; Fig. S1B] and its variant pBARGPE1-*eno* were introduced into MT27 protoplasts resulting in the strains MT27E (control strain expressing EGFP) and MT27C (expressing the enolase gene). The plasmid pBARGPE1-EGFP was acquired from the Miaolingbio Company. The pBARGPE1-*eno* plasmid was constructed by cloning the enolase gene into pBARGPE1-EGFP at the BamHI and EcoRI digestion sites (replacement of the *EGFP* sequence). The coding sequence of the enolase gene (accession number ON455119) was PCR amplified from *P. brocae* P6 cDNA using the primer pair eno-up-BamHI/eno-low-EcoRI listed in Table S1. Total RNA was isolated from *P. brocae* with a fungal RNA isolation kit (Omega Bio-Tek, Norcross, USA), and cDNA was synthesized with the PrimeScript first Strand cDNA Synthesis Kit (Takara Biomedical Technology, Beijing, China).

### AtMT of *P. brocae*

*P. brocae* was recovered and cultured on potato dextrose agar (PDA) plates at 28 °C for 7 days [warm white LED light (2000 lx) at a 16/8 h day/night cycle] until spores covered the surface of the solid medium. Spores were collected by adding liquid induction medium (IM) [consisting of minimal medium salts (2.05 g l^–1^ K_2_HPO_4_, 1.45 g l^–1^ KH_2_PO_4_, 0.15 g l^–1^ NaCl, 0.50 g l^–1^ MgSO_4_.7H_2_O, 2.0 g l^–1^ glucose, 0.1 g l^–1^ CaCl_2_.6H_2_O, 0.0025 g l^–1^ FeSO_4_.7H_2_O and 0.5 g l^–1^ (NH_4_)_2_SO_4_) supplemented with 40 mM 2-(*N*-morpholino) ethanesulphonic acid (MES) pH 5.3, 10 mM glucose, 0.5 % (v/v) glycerol and 200 µM acetosyringone] to the plate and rubbing the plate surface with a glass rod [22]. Then, the spore suspension was collected and filtered using a double layer of filter cloth. The spore number was adjusted to 10^6^ spores ml^–1^ with fresh IM and the suspension was incubated (28 °C, 160 r.p.m.) for 6 h. The binary vector pCAMBIA1303-TrpC-Hygro-gpdA-GUS-GFP was mobilized into *A. tumefaciens* AGL-1 by high-voltage electroporation. Bacteria harbouring the plasmid were incubated overnight in liquid Luria–Bertani (LB) medium containing 100 µg ml^−1^ kanamycin, and cells were collected by centrifugation (5000 r.p.m., 10 min). Then, the precipitate was resuspended with IM to OD_600_=0.3 and incubated (28 °C, 160 r.p.m.) until OD_600_=0.6 was reached. Equal volumes of spores and agrobacteria were mixed with a pipette, and 200 µl of the mixture was spread on different surfaces, namely a cellulose acetate membrane (CAM), a glass paper, a nitrocellulose membrane (NCM), a nylon membrane and a Hybond N membrane. Before co-cultivation, the sterilized membranes were placed on the surface of an IM plate containing 200 µM acetosyringone. After co-cultivation (28 °C) for 48 h, the membranes were transferred to PDA medium plates containing 300 µg ml^−1^ cefotaxime and 200 µg ml^−1^ hygromycin B to eliminate agrobacteria and non-transformed *P. brocae*, respectively. The PDA plates were incubated at 28 °C for 1 week, and *P. brocae* clones grown on the plates were picked and transferred to a new PDA plate containing 200 µg ml^−1^ hygromycin B for selection of hygromycin resistant transformants.

### Confirmation of obtained transformants

A subset of hygromycin-resistant transformants was confirmed using PCR and GUS assays. The transformants were incubated on solid Pikovskaya (PVK) medium plates containing 5 g l^–1^ Ca_3_(PO_4_)_2_ [11, 38] until spores were formed. The spores were then transferred to 10 ml of liquid PVK medium and incubated (28 °C, 160 r.p.m.) for 7 days to obtain hyphae, which were collected by centrifugation. PVK was not particularly emphasized here, other medium like PDA was also suitable. Genomic DNA was isolated from the hyphae with an HP Fungal DNA Kit (Omega Bio-Tek, Norcross, USA) and used as a template for hygromycin B resistance gene (*hph*) fragment amplification with the primers *hphF* and *hphR* (Table S1) to confirm T-DNA insertion. Furthermore, a small piece of solid PVK medium containing hyphae was taken from the edge of each clone to confirm expression of the transformed GUS gene in transformants using a GUS activity staining kit (Solarbio, Beijing, China) [38]. Transformant clones confirmed by PCR and GUS staining were stored in a 20 % (v/v) glycerol solution at −80 °C.

### Phenotype screening of mutants

To identify mutant candidates impaired in phosphate solubilization, the *P. brocae* transformants were grown on solid PVK medium plates containing 5 g l^–1^ Ca_3_(PO_4_)_2_ at 28 °C until formation of a transparent halo surrounding the clones was observed. The WT strain P6 served as a control. Mutant candidates showing a reduced halo size were selected. The halo areas were quantified with Image J software (rsb.info.nih.gov/ij).

To obtain growth curves, spores of MT27 (P6 mutant showing reduced phosphate solubilization) and the WT strain P6 (grown on PDA plates) were collected and diluted to 10^6^ spores ml^–1^. Subsequently, 5 ml of the spore suspension were inoculated into 500 ml PDB (potato dextrose broth) and the flasks were incubated at 28 °C with shaking at 160 r.p.m. The biomass (dry weight) of MT27 and WT hyphae (corresponding to 10 ml of culture medium) was determined at the second, fourth, sixth, eighth, tenth and twelfth day.

### Southern-blot analysis

To quantify the T-DNA fragment copy number in the MT27 genome, blots containing KpnI-digested MT27 genomic DNA were probed with a digoxigenin (DIG)-labelled DNA fragment amplified from pCAMBIA1303-TrpC-Hygro-gpdA-GUS-GFP (primers S1 and S2, listed in Table S1). Probe preparation, hybridization and DNA detection were performed as described in the instructions for the DIG High Prime DNA Labelling and Detection Starter Kit (Roche, Mannheim, Germany).

## TAIL-PCR

The TAIL-PCR parameters used for cloning of genomic DNA flanking T-DNA insertion sites in MT27 are shown in Table S2. The left border (LB) primers (LB1, LB2 and LB3), right border (RB) primers (RB1, RB2 and RB3) and the arbitrary degenerate (AD) primer are listed in Table S1. The relative position and melting temperature of the LB and RB primers are shown in Fig. S2. The left border flanking sequence was amplified by three tandem PCRs with the primer pairs LB1/AD, LB2/AD and LB3/AD. Likewise, the right border flanking sequence was amplified by three tandem PCRs with the primer pairs RB1/AD, RB2/AD and RB3/AD. The TAIL-PCR products were analysed by 1 % (w/v) agarose gel electrophoresis, and the highest intensity DNA bands of the tertiary reaction were excised and purified with the Majorbio Gel Purification Kit (Majorbio, Shanghai, China). Finally, the purified DNA was cloned into the PMD-18T vector (Takara, Japan) and sequenced (Sanger sequencing method).

### Phylogenetic analysis

The Basic Local Alignment Search Tool (blast) at the NCBI homepage (https://blast.ncbi.nlm.nih.gov/Blast.cgi) was used to obtain various fungal and bacterial amino acid sequences related to the protein sequence deduced from the enolase gene of P6. Alignment of these sequences was performed with Snap Gene software (version 6.0) (from GSL Biotech; available at https://www.snapgene.com). Phylogenetic analysis was conducted in mega5.0 [42] using the maximum-likelihood method based on the Tamura-Nei model [43].

### Protoplast transformation

The strains MT27C and MT27E were obtained by transformation of MT27 protoplasts with pBARGPE1-*eno* and pBARGPE1-EGFP, respectively. Vector preparation and protoplast transformation with polyethylene glycol (PEG) were performed according to the procedures described by Dias *et al*. and de Queiroz *et al*. with slight modifications [44, 45]. In total, 1×10^7^ spores were inoculated in 25 ml PDB and incubated for 36 h (28 °C, 150 r.p.m.). Mycelia were harvested by filtration using four lays of cheesecloth and washed with 0.6 M KCl. The mycelia were then suspended in lysis solution (0.6 M KCl solution, pH 5.8, containing 20 mg ml^−1^ Novozym 234) and gentle shaking for 2 h (28 °C). Mycelia debris were eliminated by filtration and the obtained protoplasts were washed four times with STC solution (1 M sorbitol, 50 mM CaCl_2_ and 100 mM Tris–HCl) by centrifugation at 2000 ***g*** for 5 min. The pellet was suspended with STC and adjusted to 10^8^ protoplasts ml^–1^. Subsequently, 0.1 ml of the protoplast suspension was mixed with 15 µg plasmid and 0.05 ml of 60 % PEG6000. The mixture was then incubated on ice for 20 min and then supplemented with 0.5 ml PEG solution. After incubation at 25 °C for 20 min, the mixture volume was adjusted to 1.5 ml with STC. The protoplasts were then collected (3000 ***g***, 10 min) and suspended in 1.5 ml regeneration media by gentle pipetting. Finally, the suspension was mixed with 20 ml of regeneration medium agar and poured onto plates. The transformants were obtained through successive rounds of bialaphos (50 µg ml^−1^; Goldbio, Saint Louis, USA) selection and single spore isolation. The transformants were PCR-confirmed by *bar* gene fragment amplification. About twelve transformants/μg DNA were obtained.

### Quantification of phosphate solubilization

To quantify the phosphate solubilization ability of *P. brocae* strains, spore suspensions (10^6^ spores ml^–1^) were pre-germinated for 24 h in PVK medium containing 5 g l^–1^ Ca_3_(PO_4_)_2_. Each suspension was then inoculated into 300 ml of PVK medium and the flasks were incubated at 28 °C with shaking at 160 r.p.m. The degree of phosphate solubilization (concentration of soluble phosphate) in culture supernatants was colorimetrically determined at 0, 24, 48, 72, 96, 120, 144 and 168 h using the Fiske and Subbarow method [46].

To explore the relationship between pH and phosphate solubilization, *P. brocae* strains were cultured in PVK or buffered PVK, both media containing 5 g l^–1^ Ca_3_(PO_4_)_2_. The pH of the (non-buffered) PVK was adjusted to 7.0 with 100 mM NaOH. The buffered PVK medium contained 100 mM Tris-HCl (pH=7.0) [47]. Then, 7 days after cultivation, the phosphate concentrations in the culture media were quantified as described above.

### Analysis of organic acids

To analyse organic acids in culture supernatants, 2 ml of each fungal suspension (grown in liquid PVK medium at 28 °C for 5 days) were centrifuged at 13 000 r.p.m. for 15 min. The pellets were used for determination of the fungal biomass (dry weight). The collected culture supernatants were passed through a 0.22 µM polytetrafluoroethylene membrane filter. The samples were then analysed in our laboratory by high-performance liquid chromatography (HPLC; Shimadzu LC-20A) with an SPD-20A detector and a CNW Athena C18-WP column (250 mm × 4.6 mm, 5 mm). The mobile phase was a 0.02 mol l^–1^ NaH_2_PO_4_ water solution (adjusted with H_3_PO_4_ to pH 2.7). The flow velocity was 0.7 ml min^−1^. Organic acids (oxalic acid, tartaric acid, malonic acid, lactic acid, acetic acid, citric acid and succinic acid) bought from Sigma-Aldrich (St. Louis, MO, USA) served as standards.

### Statistical analysis

Statistical analysis was performed with SPSS 22.0. Unless otherwise noted, differences with a *P* value≤0.05 were considered statistically significant. The halo areas of WT and MT27 strains were compared by using Student’s *t*-test. Halo areas and organic acid concentrations in culture supernatants of WT, MT27, MT27C and MT27E strains were compared using one-way ANOVA followed by Turkey’s honestly significant difference (HSD) test. The same method was also used for data analysis of culture supernatants from WT and MT27 grown in PVK and buffered PVK. The parameters were explored for normality (Shapiro–Wilk test) and homogeneity of variances (Bartlett test) prior to the one-way ANOVA. The nonparametric Kruskal–Wallis test was used to analyse effects of co-cultivation conditions on AtMT efficiency and phosphate concentrations in culture supernatants of WT, MT27, MT27C and MT27E strains.

## Results

### Optimization of AtMT and generation of a T-DNA insertion population of *P. brocae*

**Fig. 1.**
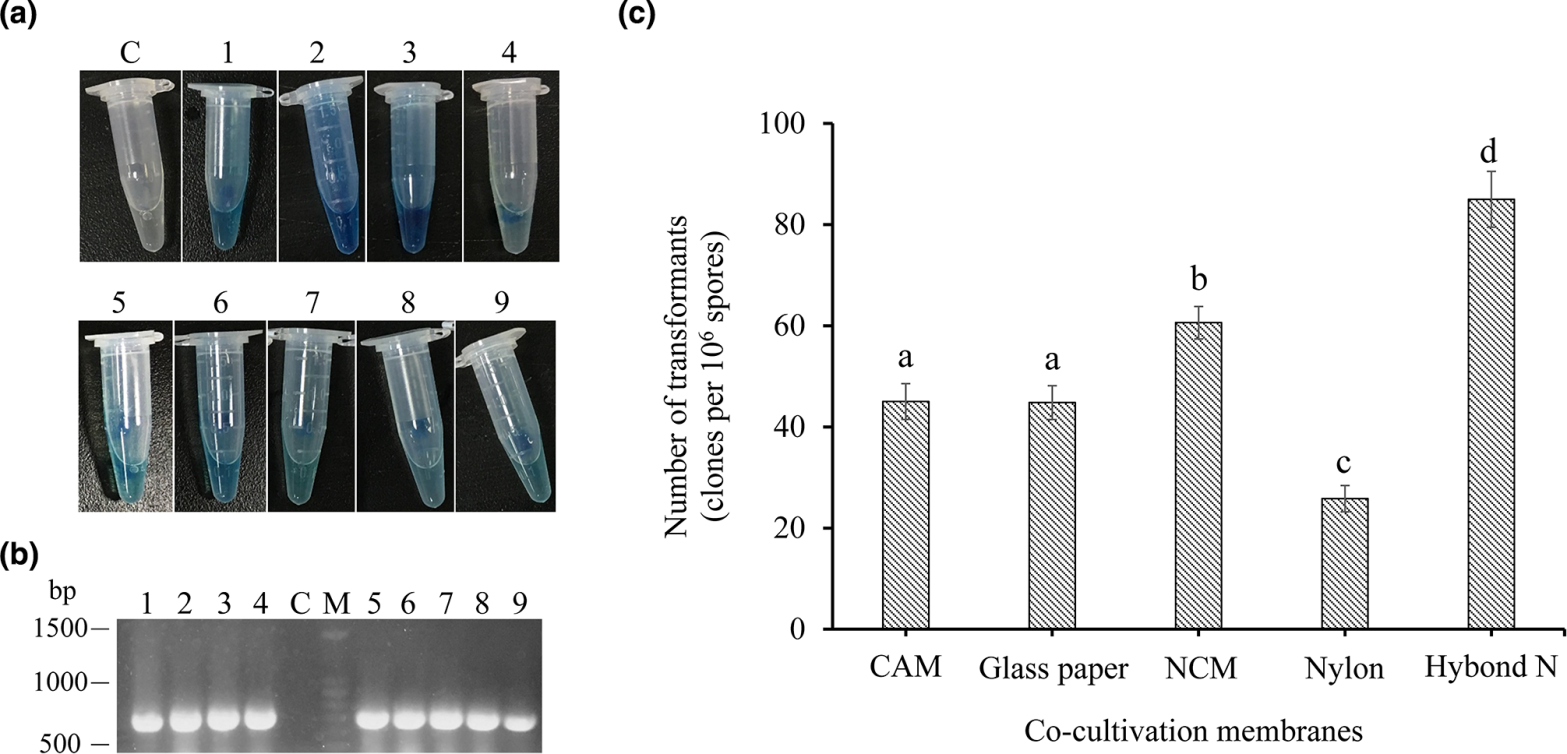
AtMT of *P. brocae* P6 and optimization of co-cultivation conditions. (**a**) Transformants showed hyphal GUS staining (blue coloration). Lane C, WT strain P6; lanes 1–9, transformants. (**b**) Agarose gel analysis of an *hph* amplicon in transformants. The PCR was performed with genomic DNA of nine different transformants (lanes 1–9). DNA of P6 was served as control template DNA (lane C); lane M, DNA marker of the agarose gel. (**c**) Optimization of co-cultivation of P6 with agrobacteria. The columns indicate the number of transformants per 10^6^ spores (means±se; *n*=5) on CAM, glass paper, NCM, nylon and Hybond N membranes. Co-cultivation on Hybond N membranes resulted in the highest transformation efficiency (different letters indicate significant differences; Kruskal–Wallis test, *P*≤0.05).

In a first experiment, AtMT of *P. brocae* P6 was performed with *A. tumefaciens* AGL-1 carrying the binary vector pCAMBIA1303-TrpC-Hygro-gpdA-GUS-GFP to test the transformation conditions established previously [38]. AGL-1 and *P. brocae* P6 were co-cultivated on glass paper and nine candidate transformants were randomly picked from the screening plates. Transformation was then confirmed by GUS staining and *hph* gene fragment amplification. As shown in Fig. 1(a, b), GUS enzyme activity was observed for all clones. Likewise, PCRs with these clones resulted in a 0.75 kb amplicon, which was confirmed to be the *hph* gene by subsequent sequencing. These results indicated that the used transformation conditions were reliable.

To further optimize the AtMT procedure of *P. brocae* P6, five kinds of co-cultivation conditions commonly used for AtMT of fungi were investigated, namely growth on CAM, glass papers, NCM, nylon membranes and Hybond N membranes. All transformants obtained in this experiment were confirmed by PCR and GUS staining. Relationships between co-cultivation conditions and AtMT efficiency are shown in Fig. 1(c). The results indicate that the material used for co-cultivation of P6 with agrobacteria had a significant influence on the obtained number of transformants. The highest yield, i.e. 85 transformants per 10^6^ spores, was obtained when Hybond N membranes were used. This is almost twice the value previously reported by our laboratory (44 transformants per 10^6^ spores) [38]. Co-cultivation of *P. brocae* and agrobacteria on NC membranes increased the transformant number by 35.3 % compared to *P. brocae* and agrobacteria co-cultivation on glass paper. Co-cultivation on glass paper and CAM led to a similar transformation efficiency, with a mean value of 45 transformants per 10^6^ spores. Co-cultivation on nylon membranes resulted in a reduced transformation efficiency, providing only 26 transformants per 10^6^ spores. Finally, taking advantage of the optimized AtMT system with Hybond N membranes, a T-DNA insertion population was generated that contained a total of approximately 4500 transformants.

### Identification and characterization of a mutant impaired in phosphate solubilization

**Fig. 2.**
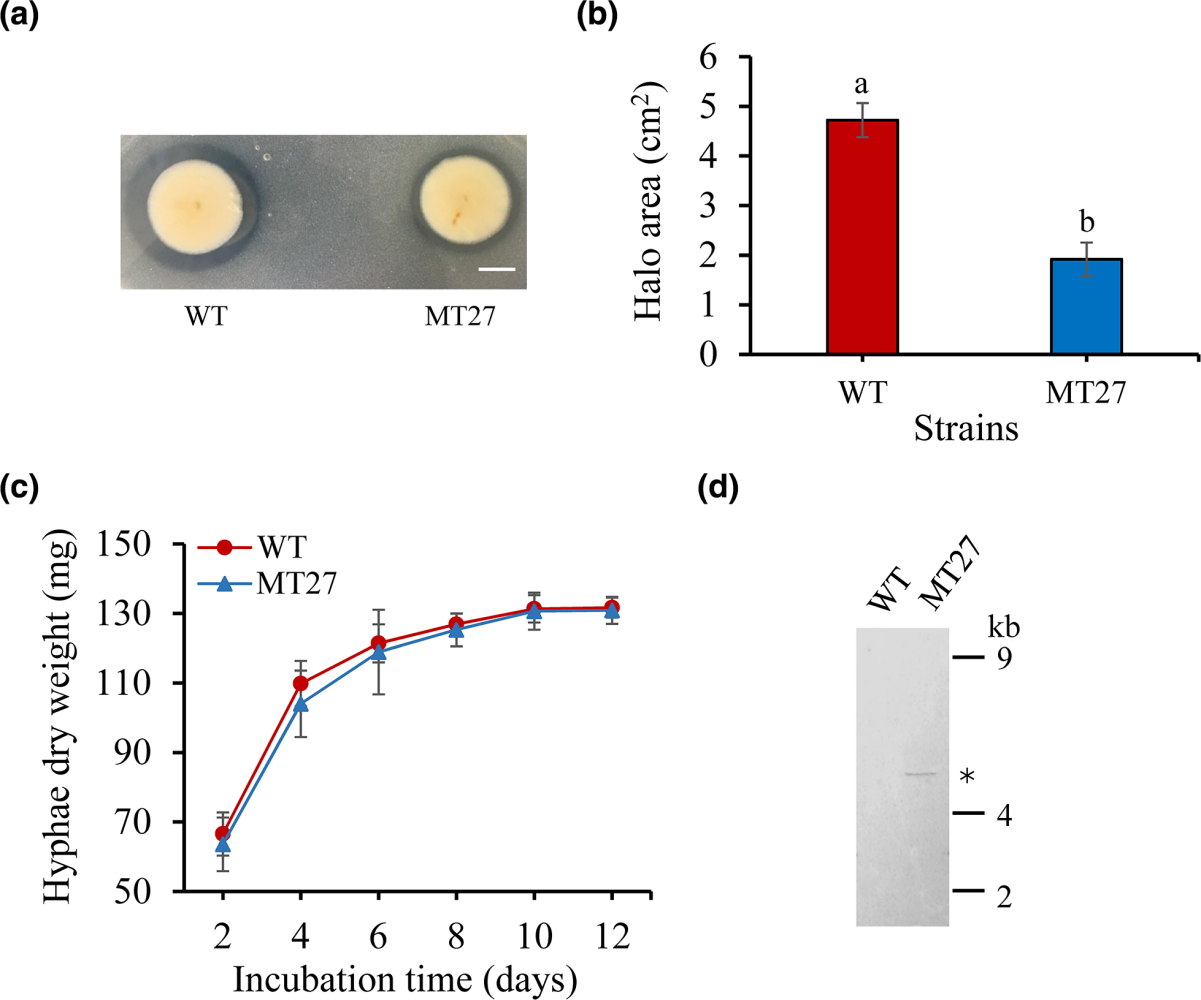
Characterization of the T-DNA insertion mutant MT27. (**a**) Phosphate solubilization phenotype of MT27 and of the WT strain P6 (WT) grown on a PVK plate containing Ca_3_(PO_4_)_2_. Scale bar=1 cm. (**b**) Quantification of the halo area for WT and MT27 colonies after cultivation on PVK plates for 5 days. Data indicate means±se (*n*=3), and different letters represent statistically significant differences (*t*-test, *P*≤0.05). (**c**) Growth curves of MT27 and WT grown in PDB. (**d**) Confirmation of a single T-DNA insertion in MT27 by Southern-blot analysis. Genomic DNA of MT27 and the parent strain (WT) was digested with KpnI. A digoxigenin-labelled probe was used to visualize the T-DNA insertion in MT27 (single band marked by an asterisk).

**Fig. 3.**
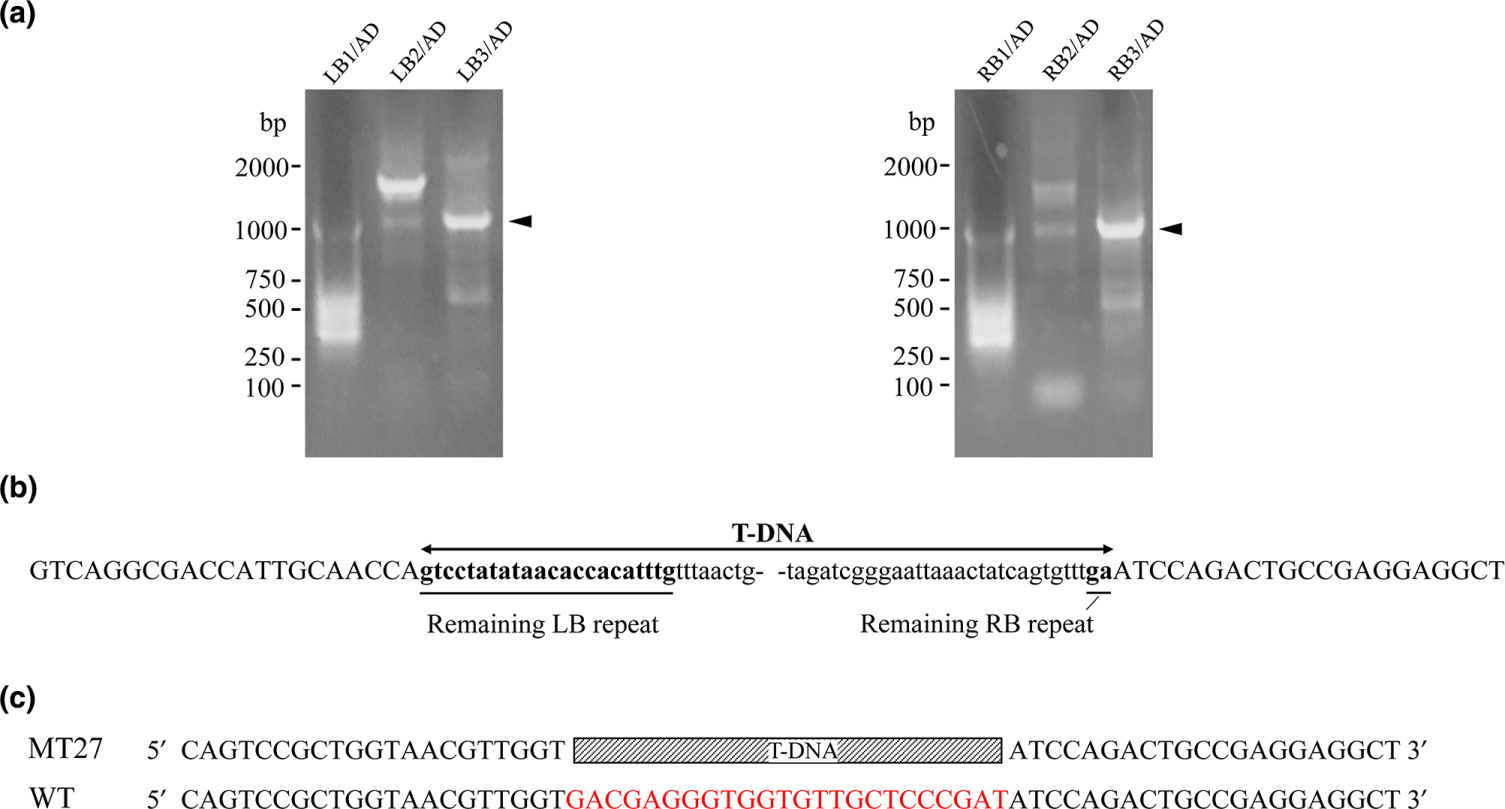
Identification of nucleotide sequences flanking the T-DNA insertion site in the MT27 genome. (**a**) Gel electrophoresis analysis of the amplicons obtained by TAIL-PCR with genomic DNA of MT27 as a template. PCR products obtained with the LB tandem PCR primers (LB1, LB2 or LB3) and AD are shown in the left panel and those obtained with the RB tandem PCR primers (RB1, RB2 or RB3) and AD in the right panel. The bands with genomic DNA flanking the T-DNA insertion site are marked by arrows. (**b**) Sequenced nucleotides flanking the T-DNA insertion site in MT27. The arrowheads indicate the 5’ to 3’ direction. The underlined and bold nucleotides correspond to the remaining RB and LB repeat sequences of the T-DNA. The capital letters indicate genomic *P. brocae* sequences flanking the T-DNA. (**c**) Alignment of genomic sequences of MT27 and WT indicated that the T-DNA insertion caused a 24 bp nucleotide loss (letters in red) in the *P. brocae* genome.

**Fig. 4.**
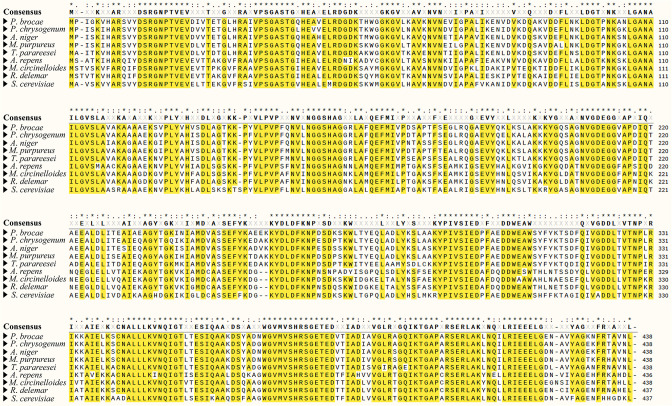
Alignment of the predicted enolase protein sequence of *P. brocae* P6 with those of seven filamentous fungi and *S. cerevisiae*. Identical amino acids are marked with an asterisk. Amino acids that match the consensus amino acid sequence (letters in bold) are highlighted with a yellow background (consensus threshold 80%). Accession numbers: USS02685.1 (*P. brocae* P6), BAC82549.1 (*P. chrysogenum* IFO 4626), XP_003188978.1 (*A. niger* CBS 513.88), TQB69604.1 (*Monascus purpureus* HQ1), OTA02066.1 (*Trichoderma parareesei* CBS 125925), ORZ23210.1 (*Absidia repens* NRRL 1336), EPB85979.1 (*Mucor circinelloides* 1006PhL), EIE87278.1 (*Rhizopus delemar* RA 99–880), NP_011770.3 (*S. cerevisiae* S288C).

**Fig. 5.**
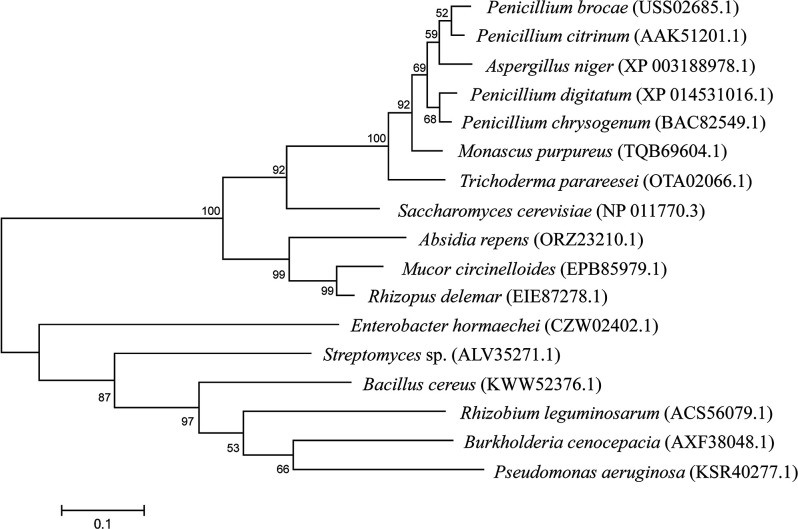
Phylogenetic tree based on selected enolase protein sequences from fungi (top) and bacteria (bottom). The scale bar indicates 0.1 amino acid substitutions per site. The numbers near the branches indicate bootstrap values (>50 %), based on parsimony analysis with 1000 replicates.

The constructed T-DNA insertion population of strain P6 was then used to screen for mutant candidates impaired in phosphate solubilization. A total of 2100 transformants from the population were inoculated on solid PVK medium with Ca_3_(PO_4_)_2_ as a phosphorus source and visually examined for their ability to form a transparent halo. Mutant candidates impaired in halo formation were selected. Among them, a mutant, named MT27, was chosen for further characterization, as it had a similar growth curve than the WT strain P6 and contained a single-copy T-DNA insertion (Fig. 2). TAIL-PCR was performed to identify the MT27 genome sequences flanking the right and left borders of the T-DNA. The obtained amplicons separated by agarose gel electrophoresis are shown in Fig. 3(a). The DNA of the obtained bands was sequenced. Nucleotide blast results showed that these sequences show highest similarity with various fungal enolase genes [e.g. 90.80 % nucleotide identity with the mRNA sequence of the enolase gene of *Aspergillus neoniger* (XM_025624297.1)]. The *P. brocae* enolase gene sequence was obtained by assembling the two sequences flanking the T-DNA (corresponding to the N-terminal and C-terminal halves of the gene). An alignment with enolase sequences from other *Penicillium* sp. allowed the prediction of the start and stop codons of the *P. brocae* enolase gene. Then, primers were designed to obtain the full-length enolase gene sequence from the P6 WT genome (GenBank accession number ON455118). Furthermore, corresponding cDNA of the enolase gene of P6 was sequenced (GenBank accession number ON455119). An alignment of these two sequences showed that the *P. brocae* enolase gene consists of four exons and three introns. Further analysis indicated that the T-DNA in the MT27 mutant was inserted into the fourth exon of the enolase gene and that the T-DNA insertion is accompanied by a 24 bp nucleotide loss compared to the WT genome (Fig. 3b, c). High sequence similarity was observed when the predicted *P. brocae* enolase amino acid sequence was aligned with published enolase protein sequences from filamentous fungi of different genera or *S. cerevisiae* (Fig. 4). Phylogenetic analysis indicated that the enolase protein sequences from fungi and bacteria show certain similarities. The sequences' similarities among fungi are greater than those among bacteria (Fig. 5). Enolase (phosphopyruvate hydratase) catalyses the conversion of 2-phosphoglycerate to phosphoenolpyruvate during glycolysis. As the reaction is reversible, enolase can also function during gluconeogenesis.

When the WT strain P6 was cultured in PVK medium containing 5 g l^–1^ Ca_3_(PO_4_)_2_, the pH of the culture supernatant was reduced. In contrast, a weaker acidification of the medium was observed for the MT27 mutant. Analysis of phosphate concentrations in culture supernatants confirmed that the MT27 mutant is impaired in phosphate solubilization. WT and MT27 strains also showed different phosphate concentrations when the growth medium was buffered with 100 mM Tris-HCl (pH=7.0). However, the measured phosphate concentrations in culture supernatants of buffered PVK were significantly lower than those obtained with PVK (Table 1). In conclusion, the obtained data indicate a possible relationship between acidification of the growth medium and the phosphate solubilization ability of *P. brocae*.

**Table 1.** pH values and soluble phosphate concentrations of culture supernatants from *P. brocae* WT strain P6 and the MT27 mutant cultured in non-buffered or buffered PVK medium Note: Data indicate means±se from three independent experiments (*n*=3). The pH of PVK containing 5 g l^–1^ Ca_3_(PO_4_)_2_ was adjusted to 7.0 with NaOH. Buffered PVK contained 5 g l^–1^ Ca_3_(PO_4_)_2_ and 100 mM Tris-HCl (pH=7.0). Different letters in the same column indicate statistically significant differences (Turkey HSD test, *P*≤0.05).

Strains	Media	pH of culture supernatant	Soluble phosphate concn (mg l^–1^)
WT	PVK	3.51±0.03 a	382.75±23.07 a
WT	Buffered PVK	4.26±0.04 b	286.32±15.14 b
MT27	PVK	4.45±0.03 c	179.64±12.53 c
MT27	Buffered PVK	5.02±0.02 d	112.51±10.06 d

### Mutant complementation

**Fig. 6.**
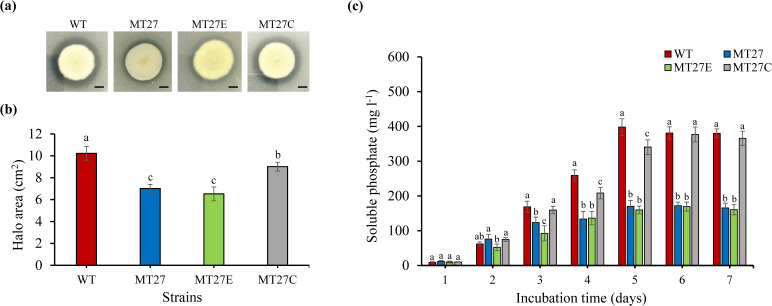
Re-expression of the enolase gene in the MT27 mutant results in increased phosphate solubilization ability. (**a**) Examples of transparent halos formed by indicated strains grown on PVK plates containing 5 g l^–1^ Ca_3_(PO_4_)_2_. Scale bar=1 cm. (**b**) Quantification of the halo area of indicated *P. brocae* strains after cultivation on the plates for 10 days. Data indicate means±se (*n*=3), and different letters represent statistically significant differences (Turkey HSD test, *P*≤0.05). (**c**) Soluble phosphate concentrations of culture supernatants from the MT27 mutant and the complementation strain MT27C. The *P. brocae* WT strain P6 and the *EGFP* expressing control strain MT27E were included into the analysis. Data indicate means±se (*n*=4), and different letters represent statistically significant differences (Kruskal–Wallis test, *P*≤0.05).

The complementation strain MT27C was constructed to confirm that the mutation of the enolase gene in the MT27 mutant is indeed related to phosphate solubilization. MT27C was obtained by transformation of MT27 with a plasmid containing the enolase gene while the control strain MT27E was transformed with an EGFP construct. The area of the transparent halo surrounding each colony was then quantified for the WT strain P6, the MT27 mutant, MT27C and MT27E. The obtained data indicated successful complementation (Fig. 6a, b). The same strains were then used to quantify their phosphate solubilization ability over time. The results showed that the concentration of soluble phosphate in the culture medium of the strains MT27 and MT27E was considerably lower than that of P6 and MT27C, respectively (Fig. 6c). These results showed that the ability to solubilize phosphate was almost completely restored in the MT27C strain and that the mutation in the enolase gene in MT27 was responsible for the poor activity to solubilize phosphate.

### The MT27 mutant secretes reduced amounts of oxalic acid and lactic acid

As the MT27 mutant showed reduced acidification of the culture medium (Table 1) and secretion of organic acids is often associated with phosphate solubilization [6], we analysed the levels of organic acids secreted by P6 and variants. The HPLC results showed that the culture supernatants contained oxalic acid, lactic acid and citric acid (Fig. S3). In contrast to the WT and the complementation strain MT27C, significantly reduced levels of oxalic acid and lactic acid were determined for the MT27 mutant and MT27E control strain. However, the concentration of citric acid in the culture supernatants did not significantly differ among the examined strains (Table 2).

**Table 2.** Levels of organic acids in supernatants of PVK medium inoculated with the *P. brocae* WT strain P6, the MT27 mutant, the control strain MT27E and the complementation strain MT27C Note: Data indicate means±se from three independent experiments (*n*=3). Levels of organic acids in culture supernatants were expressed in mg per litre culture and in mg per gram fungal biomass (DW). Different letters in the same column indicate statistically significant differences among strains (Turkey HSD test, *P*<0.05).

Strain	Biomass (mg ml^–1^)	Oxalic acid (mg l^–1^）	Lactic acid (mg l^–1^)	Citric acid (mg l^–1^)	Oxalic acid (mg g^–1^）	Lactic acid (mg g^–1^)	Citric acid (mg g^–1^)
WT	8.49±0.52	328.02±40.24 a	502.07±63.27 a	565.49±47.08 a	38.58±3.11 a	59.03±4.75 a	66.57±2.44 a
MT27	8.00±0.44	134.67±28.06 b	126.45±23.80 b	473.79±76.60 a	16.74±2.51 b	15.73±2.04 b	59.00±6.20 a
MT27E	7.58±0.82	137.47±22.85 b	130.26±12.13 b	502.97±11.99 a	18.07±1.04 b	17.21±0.37 b	66.96±8.05 a
MT27C	8.21±0.32	293.03±45.39 a	437.51±54.54 a	496.36±35.83 a	35.61±4.60 a	53.20±5.26 a	60.42±3.35 a

## Discussion

### Optimization of the AtMT system for *P. brocae*

The AtMT efficiency of fungi usually depends on various parameters such as spore concentration, co-cultivation time, concentration of acetosyringone and incubation temperature [25]. In *P. digitatum*, for example, the number of transformants per 10^6^ spores was raised from 60 to 1240 in optimization experiments [48, 49]. In our previous study, 44 *P*. *brocae* transformants per 10^6^ spores were obtained by optimization of spore concentration, acetosyringone concentration and co-cultivation time [38]. In addition, the type of the co-cultivation membrane may affect the AtMT efficiency of fungi [24, 39, 50]. For example, three different membranes (nitrocellulose, cellulose and Hybond N) were used for co-cultivation of *A. fumigatus* and *A. tumefaciens* and the best material was Hybond N, which increased the number of transformants by 2–3 times compared to nitrocellulose and cellulose [51]. In another study reporting on AtMT of *A. awamori*, it was found that the use of Hybond N or Hybond N^+^ membrane was optimal while nitrocellulose was less suitable [24]. Our *P. brocae* transformation protocol was therefore optimized by testing different co-cultivation membranes. We found that co-cultivation on a Hybond N membrane resulted in the highest transformation efficiency (85 transformants per 10^6^ spores) and that the transformation efficiency was nearly doubled compared to our previous report using glass paper [38]. We suggest that Hybond N membrane will also perform well in AtMT of other fungi. However, the molecular mechanisms underlying improved AtMT efficiency on Hybond N membranes remain unclear. It is generally assumed that filter membranes function as scaffold material to keep *A. tumefaciens* and fungal cells in close proximity [40, 51].

### Role of enolase genes

Enolase is generally known as a key enzyme in glycolysis and the gluconeogenesis pathway. There are two enolase coding genes in *S. cerevisiae*, and the encoded proteins can form homodimers and heterodimers. Mutations in the enolase genes *ENO1* or *ENO2* do not significantly impair the growth of *S. cerevisiae* and growth defects in a double-null mutant could be complemented by one of the enolase genes, indicating redundancy of enolase enzyme activity [52]. Furthermore, *S. cerevisiae* possesses additional genes encoding proteins with enolase activity [53]. Plants often possess cytoplasmic or plastidial enolase isoforms [54]. Mammals usually possess three types of enolase genes and specific forms can be used as biomarkers in cancer research [55]. The distribution and function of different enolase genes in filamentous fungi such as *P. brocae* should be investigated in future studies. The T-DNA insertion in the enolase gene of *P. brocae* did not significantly affect the growth of MT 27 in our work, suggesting that this fungus possesses at least one additional enolase gene. In general, enolase proteins have been reported to be localized in different cell compartments, such as cytoplasm, nucleus or plastids, suggesting biochemical functions besides glycolysis and gluconeogenesis [54, 56]. For example, enolase can act as a cofactor involved in tRNA targeting towards mitochondria in *S. cerevisiae* and was also found to be associated with the RNA degradosome in *E. coli* [52, 53, 57, 58]. Additionally, enolase was found on the surface of many types of cells involving bacteria, fungi and human [59]. Surface enolase is not enzymatic because it is monomeric and oligomerization is required for glycolytic activity. The *P. brocae* enolase amino acid sequence was analysed with SignalP 5.0 and no predicted signal peptide was found. Membrane-bound enolase lacks a signal peptide and the mechanism by which it attaches to the surface is unknown [59]. In the bacterium *B. cenocepacia *, a transposon mutant with a defective enolase gene showed a 34.36 % decrease in phosphate solubilization [2]. Likewise, the MT27 mutant of *P. brocae* P6 in our study was found to be impaired in phosphate solubilization due to mutation of an enolase gene.

### Relationship between enolase, secretion of organic acids and phosphate solubilization

Jiang *et al*. [60] reported that *Penicillium oxalicum* can alter accumulation of extracellular organic acids in the presence of different insoluble phosphate sources and suggested that the phosphate solubilization ability of this fungus largely depends on production of organic acids. In general, the type of carbon source of a given PSM can modulate production of organic acids and thus affect secretion of organic acids, thereby positively influencing phosphate solubilization [61, 62]. In this study, we show that MT27 is impaired in phosphate solubilization and that this mutant secretes lower amounts of oxalic acid and lactic acid compared to the WT strain P6. We suggest that reduced production of these two organic acids in MT27 leads to reduced acidification of the growth medium and weaker phosphate solubilization. MT27 contains an insertion mutation in an enolase gene and thus is impaired in production of phosphoenolpyruvate, which can be converted to pyruvate by pyruvate kinase in the glycolysis pathway. Pyruvate is an important precursor for synthesis of lactic acid and oxalic synthesis [63]. Pyruvate can be converted into lactate by the lactate dehydrogenase enzyme in the cytoplasm. Remarkably, various fungi possess a cytoplasmic pyruvate carboxylase to produce oxaloacetate from pyruvate. Oxalic acid is then synthesized from oxaloacetate by the oxaloacetate acetylhydrolase enzyme [63]. In contrast to cytoplasmic synthesis of lactic acid and oxalic acid, it is generally believed that fungi produce citric acid by the mitochondrial tricarboxylic acid cycle [64]. In our study, levels of citric acid in culture supernatants from MT27 and the WT strain were similar. Hence, the MT27 mutant appears to be specifically impaired in production of cytoplasmic organic acids derived from pyruvate. Enolase is also involved in gluconeogenesis pathways, which can synthesize glucose with precursors like lactate, glycerol and amino acids. The enzyme activity regulation pattern of *P. brocae* enolase in glycolysis/gluconeogenesis pathway still need further investigation. In conclusion, the identified enolase gene of *P. brocae* appears to be involved in synthesis of lactic acid and oxalic acid. We suggest that these organic acids, when secreted, lower the extracellular pH and act as phosphate solubilizing agents. To our knowledge, this is the first report on an enolase gene involved in phosphate solubilization in a filamentous fungus. Future experiment are needed to better understand the molecular mechanisms underlying the relationship between enolase activity, secretion of organic acids, extracellular pH and phosphate solubilization. Furthermore, it could be investigated whether the MT27 mutant is impaired in secretion of phosphatases and phytases and whether the activity of these enzymes is pH-dependent.

## Supplementary Data

Supplementary material 1
