# Supplementary material for: T-DNA insertion mutagenesis in Penicillium brocae results in identification of an enolase gene mutant impaired in secretion of organic acids and phosphate solubilization
Source: Microbiology (Reading). 2023 Apr 17;169(4):001325. doi: 10.1099/mic.0.001325 (PMC10202322; doi:10.1099/mic.0.001325)
Supplement: Supplementary material 1 [file mic-169-1325-s001.pdf]

Supplementary Tables and Figures

T-DNA insertion mutagenesis in *Penicillium brocae* results in identification of an enolase gene mutant impaired in secretion of organic acids and phosphate solubilization

Juntao Zhang, Xiaoge Han, Yang Su, Christian Staehelin and Changchao Xu

Table S1. Primers used in this study.

Table S2. Thermal and cycling conditions used for TAIL-PCR.

Fig. S1. Vectors used in this study.

Fig. S2. Information on the primers used for TAIL-PCR.

Fig. S3. Examples of HPLC chromatograms showing accumulation of organic acids in culture supernatants of *P. brocae* P6 and variants.

Table S1. Primers used in this study

Primer names	Sequence, 5' to 3'
Amplification of a hygromycin B resistant gene (hph) fragment:	
*hph*F	TACACAGCCATCGGTCCAGACG
*hph*R	CCGATTCCGGAAGTGCTTGACA
Digoxigenin-labelled DNA probe for Southern-blot analysis:	
S1	GATCTGACTAGTTTACGTCC
S2	CCCGATCTAGTAACATAG
Primers for TAIL-PCR:	
RB1	ACCAGCTCGAATTTCCCCGATCGTTC
RB2	ATTGAATCCTGTTGCCGGTCTTGC
RB3	GGGTTTTTATGATTAGAGTCCCGC
LB1	CATCTGCAATGCCTCGAGTTTCTC
LB2	CATGTGTTGAGCATATAAGAAACCC
LB3	CCGAATTAATTCGGCGTTA
AD	GNANCANAGANNGC (N = A, T, G, or C)
Cloning of the enolase gene sequence from P6 genome:	
eno-up	ATGCCTATCGGTAAGGTTCACGC
eno-low	TTACAGGTTGACAGCGGTGCG
Cloning of the coding sequence of the enolase gene from P6 cDNA:	
eno-up-BamHI	CGGGATCCATGCCTATCGGTAAGGTTCACGC
eno-low-EcoRI	CGGAATTCTTATTACAGGTTGACAGCGGTGCG

Table S2. Thermal and cycling conditions used for TAIL-PCR

Reaction	File no.*	Cycle no.	Thermal conditions
Primary	1	1	94 ^{o}C (2 min), 95 ^{o}C (1 min).
	2	5	94 ^{o}C (15 s), 65 ^{o}C (1 min), 72 ^{o}C (2 min).
	3	1	94 ^{o}C (15 s), 30 ^{o}C (3 min), ramping to 72 ^{o}C (0.2 ^{o}C/s), 72 ^{o}C (2 min).
	4	10	94 ^{o}C (5 s), 45 ^{o}C (1 min), 72 ^{o}C (2 min).
	5	12	94 ^{o}C (5 s), 65°C (1 min), 72 ^{o}C (2 min);<br>94 ^{o}C (5 s), 65°C (1 min), 72 ^{o}C (2 min);<br>94 ^{o}C (5 s), 45°C (1 min), 72 ^{o}C (2 min).
	6	1	72 ^{o}C (2 min).
Secondary	7	15	94 ^{o}C (5 s), 65°C (1 min), 72 ^{o}C (2 min);<br>94 ^{o}C (5 s), 65°C (1 min), 72 ^{o}C (2 min);<br>94 ^{o}C (5 s), 45°C (1 min), 72 ^{o}C (2 min).
	6	1	72 ^{o}C (2 min).
Tertiary	8	20	94 ^{o}C (15 s), 45°C (1 min), 72 ^{o}C (2 min).
	9	1	72 ^{o}C (5 min).

* The program files in each reaction were linked automatically.

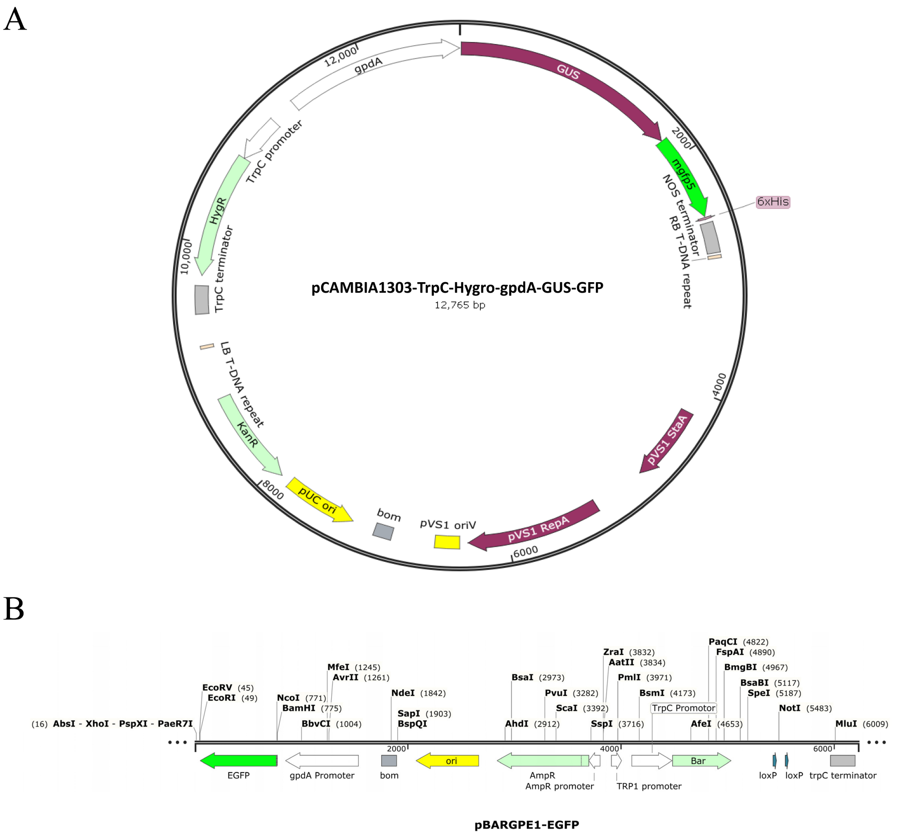

Fig. S1. Vectors used in this study. (A) Circled pCAMBIA1303-TrpC-Hygro-gpdA-GUS-GFP vector map. (B) Linearized pBARGPE1-EGFP vector map showing single restriction endonuclease digestion sites. The map was generated by Snap Gene version 6.0.2 (https://www.snapgene.com).

A

RB1, Tm = 65.7 ℃ RB2, Tm = 63.0 ℃

gtgaccagctcgaatttccccgatcgttcaaacatttggcaataaagtttcttaagattgaatcctgttgccggtcttgc 80

gatgattatcatataatttctgttgaattacgttaagcatgtaataattaacatgtaatgcatgacgttatttatgagat 160

RB3, Tm = 58.5 ℃

gggtttttatgattagagtcccgcaattatacatttaatacgcgatagaaaacaaaatatagcgcgcaaactaggataaa 240

ttatcgcgcgcggtgtcatctatgttactagatcgggaattaaactatcagtgttt**gacaggatatattggcgggtaaac** 320

RB T-DNA repeat (24 bp)

B

LB1, Tm = 66.5 ℃

cgggtttacctcttccagatacagctcatctgcaatgcctcgagtttctccataataatgtgtgagtagttcccagataa 80

LB2, Tm = 63.2 ℃

gggaattagggttcctatagggtttcgctcatgtgttgagcatataagaaacccttagtatgtatttgtatttgtaaaat 160

LB3, Tm = 58.5 ℃

acttctatcaataaaatttctaattcctaaaccaaaatccagtactaaaatccagatccccgaattaattcggcgtta 240

attcagtacattaaaaacgtccgcaatgtgttattaagttgtctaagcgtcaattt**gtttacaccacaatatatcctgcc** 320

LB T-DNA repeat (24 bp)

C

AD, Tm = 47.5 ℃: GNANCANAGANNGC (N = A, T, G, or C)

Fig. S2. Information on the primers used for TAIL-PCR. (A) Right border (RB) end of the T-DNA showing the sequences and melting temperature (Tm) information of the tandem primers RB1, RB2 and RB3 used in TAIL-PCR. (B) Left border (LB) end of the T-DNA showing the sequences and Tm information of the tandem primers LB1, LB2 and LB3 used in TAIL-PCR. The bold letters in (A) and (B) indicate T-DNA repeats and the region between T-DNA border repeats is transferred to fungi cells. (C) Information on the arbitrary degenerate primer (AD) used in TAIL-PCR.

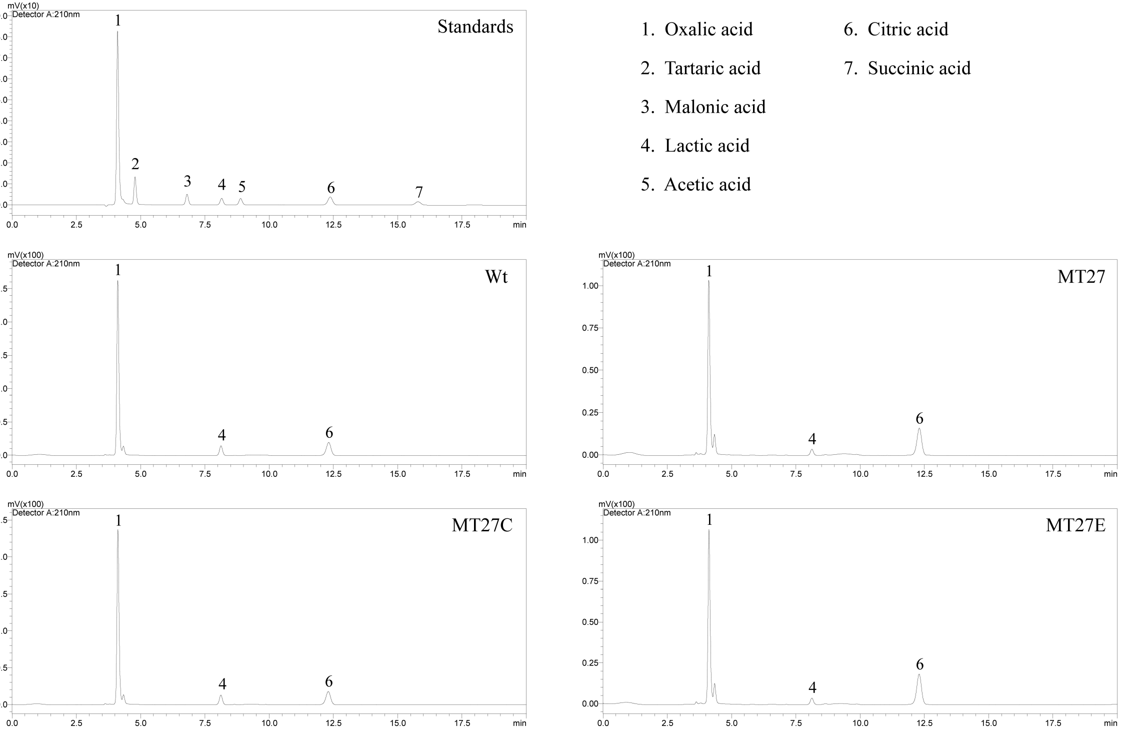

Fig. S3 Examples of HPLC chromatograms showing accumulation of organic acids in culture supernatants of *P. brocae* P6 and variants. (Note: The Y- axis has variable scales for each chromatogram).
